# The Microbiome Associated with the Reef Builder *Neogoniolithon* sp. in the Eastern Mediterranean

**DOI:** 10.3390/microorganisms9071374

**Published:** 2021-06-24

**Authors:** Shany Gefen-Treves, Alexander Bartholomäus, Fabian Horn, Adam Boleslaw Zaborowski, Dan Tchernov, Dirk Wagner, Aharon Oren, Aaron Kaplan

**Affiliations:** 1 Department of Plant and Environmental Sciences, Edmond J. Safra Campus, The Hebrew University of Jerusalem, Givat Ram, Jerusalem 9190401, Israel; shany.gefen@mail.huji.ac.il (S.G.-T.); aharon.oren@mail.huji.ac.il (A.O.); 2 GFZ German Research Centre for Geosciences, Section Geomicrobiology, 14473 Potsdam, Germany; abartho@gfz-potsdam.de (A.B.); gfz.fabian@gmail.com (F.H.); dirk.wagner@gfz-potsdam.de (D.W.); 3 Bioinformatics Group, Max Planck Institute for Molecular Plant Physiology, 14476 Potsdam-Golm, Germany; Zaborowski@mpimp-golm.mpg.de; 4 Leon H. Charney School of Marine Sciences, University of Haifa, Haifa 3498838, Israel; dtchernov@univ.haifa.ac.il; 5 Institute of Geosciences, University of Potsdam, 14476 Potsdam, Germany

**Keywords:** microbiome, reef builder, 16S rRNA sequencing, algal, bacteria, interaction

## Abstract

The development of coastal vermetid reefs and rocky shores depends on the activity of several reef builders, including red crustose coralline algae (CCA) such as *Neogoniolithon* sp. To initiate studies on the interaction between *Neogoniolithon* sp. and its associated bacteria, and their impact on the algae physiological performance, we characterized the bacterial community by 16S rRNA gene sequencing. These were extracted from the algal tissue and adjacent waters along two sampling campaigns (during winter and spring), in three study regions along a reef in the east Mediterranean Israeli coast and from laboratory-grown algae. The analysis revealed that aquaria and field communities differ substantially, suggesting that future research on *Neogoniolithon* sp. interaction with its microbiome must rest on aquaria that closely simulate coastal conditions. Some prokaryote classes found associated with the alga tissue were hardly detected or absent from surrounding water. Further, bacterial populations differed between sampling campaigns. One example is the presence of anaerobic bacteria and archaea families in one of the campaigns, correlating with the weaker turbulence in the spring season, probably leading to the development of local anoxic conditions. A better understanding of reef-building activity of CCA and their associated bacteria is necessary for assessment of their resilience to climate change and may support coastal preservation efforts.

## 1. Introduction

Vermetid reefs along the warm ultra-oligotrophic eastern Mediterranean coast constitute a hotspot of biodiversity and ecosystem functioning. Despite the prevailing harsh natural conditions, including hydration–desiccation cycles, mechanical stress, rapid pH shifts, and fluctuating light intensity [1], these reefs, consisting of structures termed abrasion platforms [2], are among the most important biogenic habitats of temperate waters. They play a major role in preventing coastal erosion, altering the sediment and nutrient transport, and providing a habitat for a variety of fish, invertebrates, and algae [2,3,4,5]. These ecosystem engineering functions are largely attributed to two species of intertidal vermetid gastropods [2] and to crustose coralline algae (CCA) such as *Neogoniolithon brassica-florida*, which cements the reef structures [5,6]. Coralline algae are ubiquitous key ecosystem engineers in temperate rocky shores, including in the Mediterranean Sea. Further studies are needed to determine who is there and assess their abundance [7]. Cementing is achieved through deposition of calcareous thalli, giving rise to various bioconstructions such as coralligenous concretions, rims, and beds (see [7,8,9], and references therein). However, the engineering role played by CCA extends beyond the macro-scale. The balance between inorganic carbon uptake, photosynthesis, calcification, and decalcification [1,10] has been shown to create temporal and spatial variability in pH, alkalinity, and specific ion availability on the CCA surface [11,12,13]. These processes create an altered microenvironment in the immediate surrounding of the algae by affecting hydrology, mineralogy, carbohydrate composition, and pH that may support the development of microbial communities. These communities are distinct from those prevailing in the surrounding seawater [14,15,16] and may contribute to the function and diversity of the reefs.

**Figure 1:**
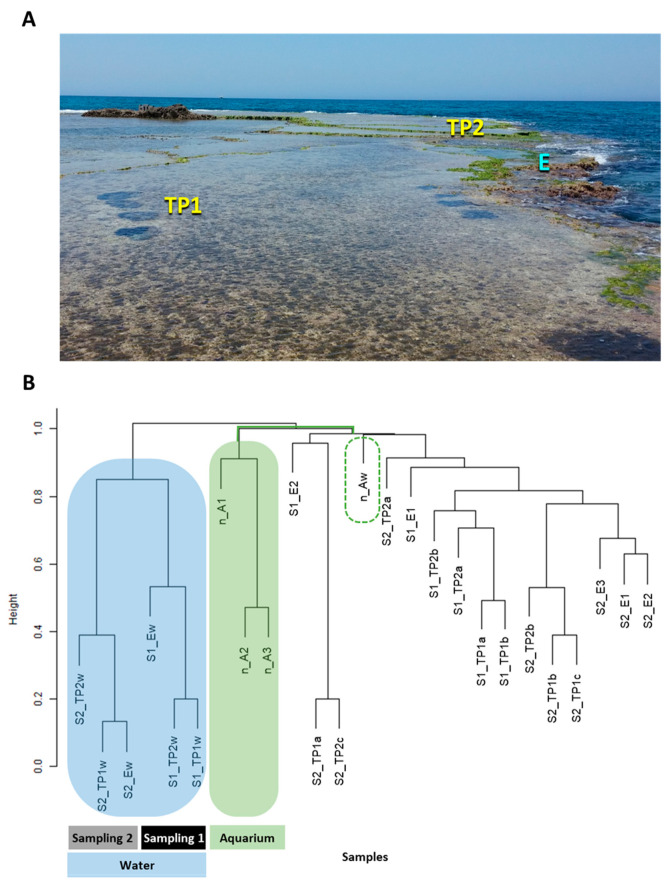
(**A**) Study sampling site in the Sdot-Yam abrasion platform. Study regions included tidal pools (TP, 1 and 2) and platform edge (E). (**B**) Hierarchical clustering of samples from field- and aquaria-grown *Neogoniolithon* sp. surfaces and the seawater surrounding the natural population. S1 = sampling 1, S2 = sampling 2, TP (1 and 2) = tidal pools, E = platform edge, w = water sample, a, b, c = biological replicates. Note the branching of aquaria samples from both algal surface and water clusters.

Similar to other organisms and habitats, the CCA-associated microbiomes are implicated with host health [17], metabolism [18], and reproduction [15]. Thus, changes in the microbiome composition may serve as an indicator of biotic [17] and abiotic [19,20] stresses, may be applied as a useful tool in the design of coastal preservation efforts [18], and as a measure for CCA responses to long-term climate change [7,17]. Most of our knowledge on the importance of host–microbe relationships rests on studies on tropical CCA species [15]. However, despite the importance of CCA for Mediterranean vermetid reefs and the need to monitor their function [1], studies characterizing their associated microbiome in the Mediterranean ecosystem are scarce [7]. Examination of reef-building CCA tissue withdrawn from the abrasion platforms along the eastern Mediterranean shore in the reefs of Sdot-Yam, Israel (Figure 1A), illustrated the tight association between bacterial cells and algal surface (Figure S1). Here, we initiated studies on the composition and potential functions of the natural microbial community associated with reef-building CCA identified as *Neogoniolithon* sp. along these reefs, as compared with their surrounding water and with samples collected from the same site and grown in controlled aquaria. Our results show distinct differences between the prokaryote compositions associated with the *Neogoniolithon* sp. surface and shed light on potential interspecies interactions.

## 2. Materials and Methods

### 2.1. Sample Collection and Processing 

Samples of rocks bearing CCA (using hammer and chisel) and their surrounding seawater (see below) were collected from an eolianite vermetid reef on the eastern Mediterranean shore, Sdot-Yam, Israel (32°29′38.61″ N, 34°53′16.65″ E; Figure 1A), during sampling campaigns 1 and 2 (January 2017 and May 2018, respectively). Due to the wide seasonal range of abiotic conditions (including ~15 °C temperature amplitude [21] and vast precipitation differences (3.7 vs. 131 mm monthly average; https://ims.data.gov.il/, accessed on 14 March 2021)) in these reefs, sampling campaigns were timed to the winter (sampling 1) and spring (sampling 2). In addition, the anthropogenic activities on or nearby the reefs varies between these seasons, as Sdot-Yam abrasion platforms are a popular recreation site during spring and summer, including fishing, maritime sports, and sailing activities from a small marina. Based on morphological characteristics [22], the CCA samples were originally identified as *Neogoniolithon brassica-florida*, but since we were unable to characterize each one of them, we referred to them as *Neogoniolithon* sp.

In this study, tide pools and platform edges are defined as different “study regions” on the reef of Sdot-Yam (Figure 1A). Each study region may create a distinct biological niche, possibly differentially affected by environmental conditions (e.g., exposure to waves, light, and temperature shifts), impacting the microbial diversity.

The samples were collected from three study regions on the reef—two tidal pools—TP1 (eastern, closer to shoreline) and TP2 (western, closer to open sea), about 10 m apart from one another, and the platform edge (Figure 1A). Biological independent replicates were sampled in each study region (*n* = 2 in sampling 1 and *n* = 3 in sampling 2) from various parts in the tidal pools and the platform edge, making sure that different individuals were sampled in each site. Sampling was performed as described in [15] with several modifications. Briefly, water samples (*n* = 3) were taken from each CCA study region using a 30 mL sterile syringe by opening the syringe underwater, rinsing 3 times, and then closing before removal from the water column. Samples were kept on ice until processing (<3 h). Under sterile conditions in the lab, seawater samples were pooled and filtered through a 0.22 µm sterile filter (Millex-GV, 33 mm, Merck Chemicals, Darmstadt, Germany) and then filters were removed into sterile tubes and stored at −80 °C.

CCA-covered rocks were thoroughly rinsed under a stream of 30 mL of filtered (through 0.22 μm filters) artificial sea water (ASW; Red Sea Coral Pro Salt in filtered de-ionized water to 36 PSU, pH 8.2) to remove excessive particulate matter. Samples were taken by scraping the surface (1–3 cm^2^) of CCA-covered rocks using a sterilized scalpel blade, thereby collecting algal tissues as well as microbial communities. Tissues containing scalpel blades were placed in 1 mL tubes and frozen immediately in liquid nitrogen until further treatment.

Next, 1 mL of filtered ASW was added to each tube containing either seawater sample filters or scalpel blades, followed by vigorous shaking. Suspensions were centrifuged for 25 min at 13,000× *g* and the pellet was stored at −80 °C.

*Neogoniolithon* sp.-covered rocks were collected from various places in the site shown in Figure 1A and placed in aquaria for future host–microbiome interaction studies. For microbial community analysis of aquaria samples, these algal-covered rocks were thoroughly rinsed with ASW to remove excessive particulate matter and epifauna, with the help of tweezers, under a dissecting scope (Kyowa optical, SDZ-PL, Tokyo, Japan). This procedure was repeated monthly as a maintenance treatment. Rocks were kept in glass aquaria circulated with ASW through naturally developed biological filters (on synthetic sponges and ceramic beads) for over a year prior to the analyses described here, to allow enough time for biomass accumulation of these slow-growing algae. Growth conditions were 25 °C, 30 µmol photons m^−2^ s^−1^ at the surface with 12:12 h light/dark cycles using a LED array (warm white, 3000 °K, SMD 3528 600Leds). Growth tanks were bubbled with air at ~200 mL min^−1^ [1]. Algal tissue from aquaria algal-covered rocks (*n* = 3) was collected and processed as described above for fresh rocks.

### 2.2. Sequencing

Microbial DNA was extracted using a PowerSoil DNA isolation kit (MO BIO, Jefferson City, MO, USA). Microbial community analysis was based on high-throughput sequencing of 16S rRNA gene amplicons designed to amplify the relevant prokaryote sequences using paired-end 16S community sequencing on the Illumina platform. Extracted DNA samples were sent to Hylabs (Rehovot, Israel) for sequencing. Each sample was tagged with a unique barcode combination and 515F [23] and 806R [24] modified primers, 515F 5′-GTGYCAGCMGCCGCGGTAA-3′, 806R 5′-GGACTACNVGGGTWTCTAAT-3′ for the 16S SSU rRNA V4 region, following the Earth Microbiome Project (EMP) guidelines (https://earthmicrobiome.org/, accessed on 1 December 2019).

Samples were cleaned using AMPure beads and checked for DNA concentration and size via Qubit and TapeStation. Samples were then loaded on the Illumina MiSeq for a paired-end run and raw reads were de-multiplexed using the Illumina MiSeq software. Sequencing data were deposited in NCBI public domain (accession numbers—BioProject: PRJEB38881, BioSamples: ERS4666887-ERS4666911).

### 2.3. Data QC and Analysis

Sequencing raw reads were adapter and quality trimmed using cutadapt v2.8 using pair-end mode and following parameters: −e 0.2 −q 15,15 −m 150 −discard-untrimmed. ASVs were generated using trimmed reads and DADA2 package v1.10.1 [25] using the pooled (function call dada (…, pool = TRUE)) approach following parameters: truncLen = c(200,150), maxN = 0, rm.phix = TRUE, compress = TRUE, multithread = TRUE, minLen = 150 with R v3.6. Taxonomic assignment was conducted using DADA2 and SILVA database v138. The number of inputs, processed, and final reads of the DADA2 pipeline are shown in Table S1. Subsequently, ASVs representing chloroplasts and mitochondria were removed.

The data processing procedure described above resulted in two different tables: (i) A rarefied table with integer counts for each of the technical and biological replicates. Rarefaction analyses were generated to ensure that a sufficient number of reads were obtained for each sample (Figure S2). They were performed with sample size *n* = 3000 as this is the minimum resulting reads (see Table S1). (ii) A table with relative abundance where technical replicates are averaged. This table was generated by: first, removing singletons; second, calculating relative abundance; third, technical replicates were averaged by arithmetic mean; fourth, rare biosphere <0.1% relative abundance was removed. The rare biosphere was removed to avoid overinterpretation of very low abundance species [26,27,28].

Microbial community analysis was performed using R v3.6, with the following packages: phyloseq 1.26.1 [29], vegan 2.5.6. Alpha diversity measures were calculated on the rarefied table, significance was calculated using ANOVA and Tukey’s Honest Significant Difference method. NMDS plots are based on the relative abundance table and the Bray–Curtis dissimilarity. PERMANOVA to compare microbial communities on ASV level was performed using Bray–Curtis dissimilarity using the adonis function of the vegan package. Bubble plots were generated using the processed relative abundance with the help of custom and public functions provided: https://github.com/AlexanderBartholomaeus/BubblePlot accessed on 16 March 2021. Bar charts and dendrograms are based on the relative abundance table. All bubble, bar, 2D scatter, and box plots excluding the Venn diagrams (VennDiagram v1.6.20) and dendrogram (R base, hclust plot) were generated using ggplot2 v3.3.0 plotting library in R. The dendrogram was generated using Pearson correlation and average linkage clustering. The scripts and tables for the data analysis, processing, and generation of the plots are provided at github: https://github.com/AlexanderBartholomaeus/ReefBuilderMicrobiome accessed on 16 March 2021. Unique and shared taxa were identified by manual inspection of the data.

Functional annotation of ASV was generated using FAPROTAX 1.2.3 complete package [30] using the “collapse_table.py” script against the FAPROTAX database (http://www.loucalab.com/archive/FAPROTAX, accessed on 16 March 2021). Final functional annotation values were obtained by multiplying ASV abundance with presence/absence functional annotation. Since one ASV can have multiple functions, summed functional annotation for each sample may be larger than 1.

## 3. Results

### 3.1. Neogoniolithon sp. Hosts a Distinct Prokaryotic Community on Its Surface

We analyzed the composition of the microbial community within samples withdrawn from the surfaces of *Neogoniolithon* sp. as compared with those in the surrounding water. With this being an exploratory study and to minimize damage to the protected tidal pools within abrasion platforms, we limited our sampling to three close-by study regions (Figure 1A) during two sampling campaigns, in January 2017 and May 2018 (hereafter, sampling 1 and 2, respectively, see Section 2).

After the removal of low abundant amplicon sequence variants (ASVs, see Section 2), the tissue surface and water samples from *Neogoniolithon* sp. inhabiting the reef and that collected from aquaria-derived samples were clustered. These were the first data sets confirming the development of distinct communities on the algal surfaces in each habitat (Figure 1B). All the algal samples branched separately from their surrounding water samples. Further, and interestingly, the patterns observed also revealed a clear separation between reef- and aquarium-grown samples, likely because the latter were kept under constant laboratory conditions that differ from those in the reef. Different study regions and sampling campaigns demonstrated partially overlapping clustering with no unique branching.

**Figure 2:**
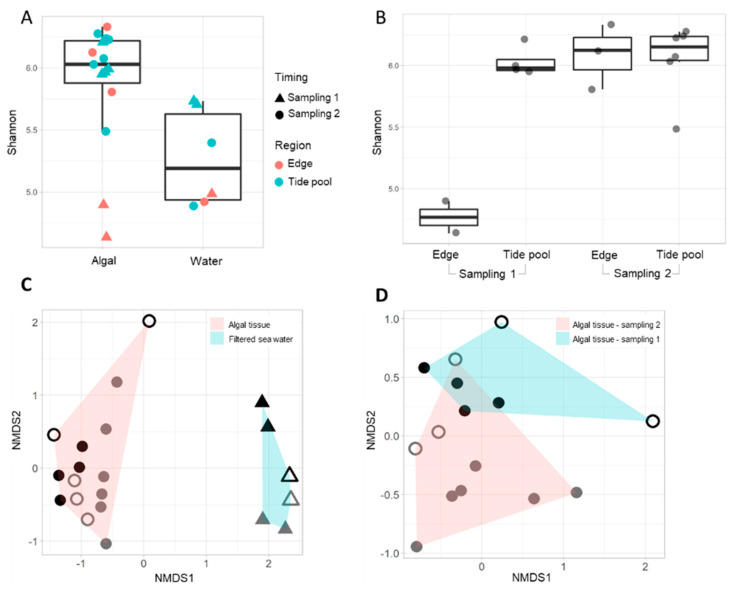
(**A**,**B**) Alpha diversity analyses calculated using the Shannon index of *Neogoniolithon* sp. surfaces and their surrounding water (**A**) in sampling 1 (triangles) and sampling 2 (circles), in different study regions (tidal pools, turquoise; platform edge, red). Water was collected from the same study region where the algal samples were collected. Significance was calculated using ANOVA and Tukey’s Honest Significant Difference method, *p* value = 0.0155. (**B**) Alpha diversity analysis of algal samples in tidal pools and platform edge. Significant differences were observed only for sampling 1 tidal pool–Edge (*p* value = 4.8 × 10^−4^) and sampling 1–Edge–sampling 2–Edge crossings (*p* = 9.2 × 10^−3^). Other alpha diversity indices demonstrated similar trends, see Figure S3. (**C**) NMDS analyses with Bray–Curtis distance (stress value 0.091) for algal and water samples. A significant difference between community structure of algal and water samples was observed (PERMANOVA, *p* value = 0.001). (**D**) NMDS analyses with Bray–Curtis distance (stress value 0.1104) for algal samples in samplings 1 and 2. Samplings 1 and 2 also demonstrate distinct community structures (*p* value = 0.002). (**C**,**D**) Circles, algal samples; triangles, water samples; closed symbols, tide pools; empty symbols, edge; during sampling 1 (black) and sampling 2 (grey).

There are clear indications that the *Neogoniolithon* sp. surfaces-associated microbiome differs from that present in the surrounding water. First, alpha diversity values were higher in algal samples compared to water samples, indicating that the algal communities are more diverse (*p* value = 0.0155, Figure 2A and Figure S3). Algal-associated communities from different samplings could not be clearly distinguished by alpha diversity analyses, with the exception of samples from the platform edge region taken during sampling 1 (Figure 2B). Second, a clear separation between the algal-associated and water sample communities is also indicated by the beta diversity analyses (*p* value = 0.001, i.e., non-metric multidimensional scaling, NMDS, Figure 2C). Significant differences could also be observed between sampling 1 and sampling 2 (*p* value = 0.002, Figure 2D). In addition, samples from aquaria demonstrated larger variance in their alpha diversity, which was generally lower compared to most field algal samples (Figure S4). Examination of the similarity between specific algal tissue samples and their surrounding water at the ASV level revealed only a 2.2–9.0% overlap (Figure S5A), compared with 25–31% between algal samples and 44–72% between the well-mixed water samples (Figure S5B).

**Figure 3:**
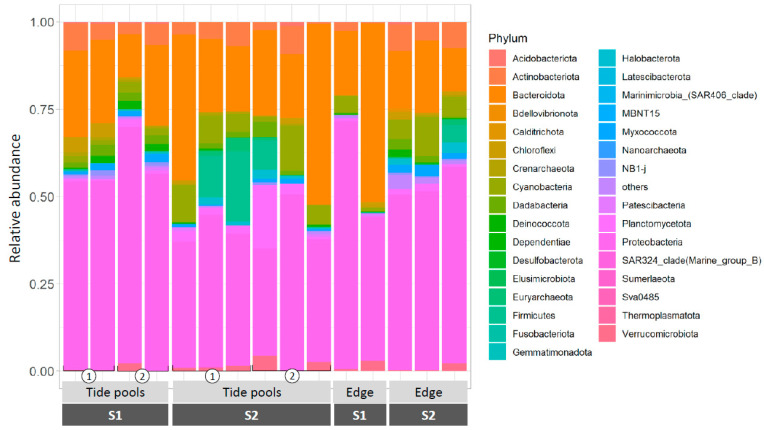
Relative abundance of ASVs associated with *Neogoniolithon* sp., phylum-level. Rock samples were taken from the platform edge and tidal pools during sampling 1 (S1) and sampling 2 (S2), followed by DNA extraction of suspensions of *Neogoniolithon* sp.-scraped tissues. Values represent the fraction from total ASVs identified in each sample. Asterisks denote samples demonstrating composition variance compared with their associated replicates.

Prokaryote samples withdrawn from natural *Neogoniolithon* sp. surfaces were dominated by Proteobacteria (49.09 ± 11.66%), Bacteroidota (25.22 ± 12.64%), Cyanobacteria (5.33 ± 3.83%), and Actinobacteriota (4.73 ± 2.60%), (Figure 3, the complete list is provided in Table S2). Those were present in all the samples tested, regardless of time of sampling and location, and are shown in Figure 3. Therefore, they are hereby defined as the core community associated with *Neogoniolithon* sp. In most cases, the community composition was largely reproducible across biological replicates. However, in some cases, a larger variation was observed (see asterisks in Figure 3). This could result from differences in the local microenvironment conditions but might also be due to sampling of a different *Neogoniolithon* species.

**Figure 4:**
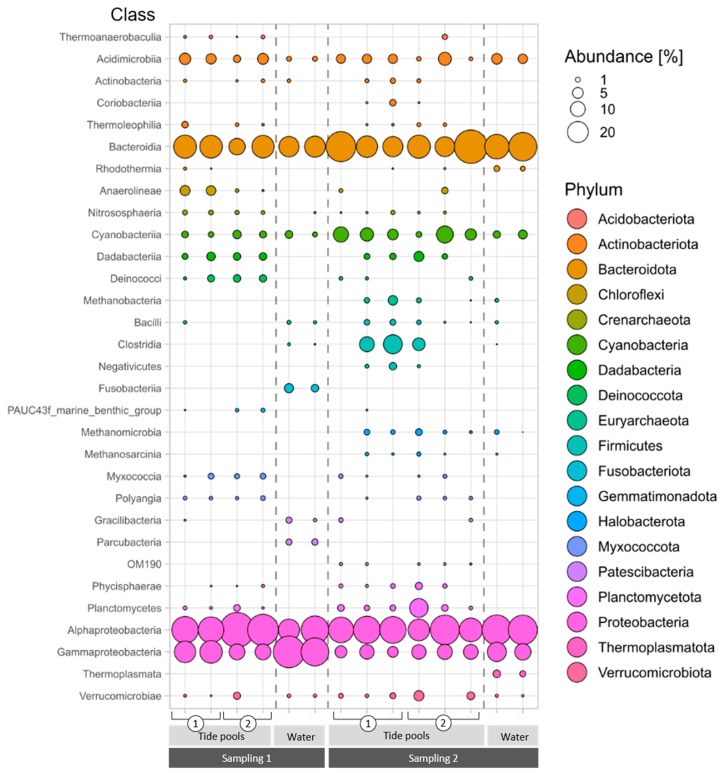
Relative abundance of bacterial ASVs associated with *Neogoniolithon* sp. surfaces in class taxonomic level, from tidal pool and water samples in sampling 1 (*n* = 2) and sampling 2 (*n* = 3). Colors indicate the various phyla that the classes belong to. Bubble size represents the abundance in percentage of each class (see scale). For family level analysis, see Figure S6.

Since each of these phyla contains organisms that may differ substantially in their functional characteristics, the latter was also examined at the class and family levels. Within the phyla identified in Figure 3, most ASVs belonged to the classes Alphaproteobacteria (35.21% ± 11.13%), Bacteroidia (25.15 ± 12.66%), Cyanobacteriia (5.32 ± 3.81%), and Acidimicrobiia (3.84 ± 2.32%) (Figure 4). The complete list of classes belonging to the core community (the four main phyla, Figure 3) is provided in Table S3. The analysis revealed that some classes were detected in the algal but not in the water samples (Figure 4). This include Dadabacteriia (Dadabacteria), Thermoleophilia (Actinobacteriota), Myxococcia (Proteobacteria), Polyangia (Proteobacteria), Phycisphaerae and Planctomycetes (Planctomycetes), Anaerolineae (Chloroflexi), and Deinococci (Deinococcota).

Further, the analysis revealed that some of the classes were present in all the samples withdrawn regardless of timing and study region. Examples (the more abundant) include Alphaproteobacteria, Gammaproteobacteria, Bacteroidia, Acidimicrobiia, and Cyanobacteriia (Figure 4). Others showed a distinct temporal and spatial presence. As an example, samples withdrawn from *Neogoniolithon* sp. within the tidal pools in sampling 2, but not in sampling 1, were characterized by the presence of Methanobacteria, Methanomicrobia and Methanosarcinia (Euryarchaeota), Clostridia and Negativicutes (Firmicutes), OM190 (Planctomycetes), and Coriobacteriia (Actinobacteriota). Others, such as Planctomycetes and Phycisphaerae (Planctomycetes) were present in a higher abundance in sampling 2 compared to sampling 1 (Figure 4). Those were all missing in the samples withdrawn from the well-aerated aquaria.

Sampling 1 samples were richer in Deinococci (Deinococcota) and Anaerolineae (Chloroflexi). Of particular interest are those families that were abundant in the algal tissue as compared with their surrounding water (Figure S6). Sampling 2 tidal pools included sequences related to anaerobic bacteria belonging to Lachnospiraceae and Ruminococcaceae (Clostridia), Veillonellaceae (Negativicutes), Enterococcaceae (Bacilli), as well as aerobic bacteria such as Rikenellaceae and Barnesiellaceae (that belong to Bacteroidota—also known as Bacteroidia and Bacteroidetes ([31], Figure S5). Those were not detected in either sampling 1 or the surrounding water. In contrast, the classes Fusobacteriia (Fusobacteriota) and Parcubacteria (Patescibacteria) were present only in water samples collected in sampling 1 (Figure 4).

### 3.2. Functional Analysis of the Neogoniolithon sp.-Associated Community

**Figure 5:**
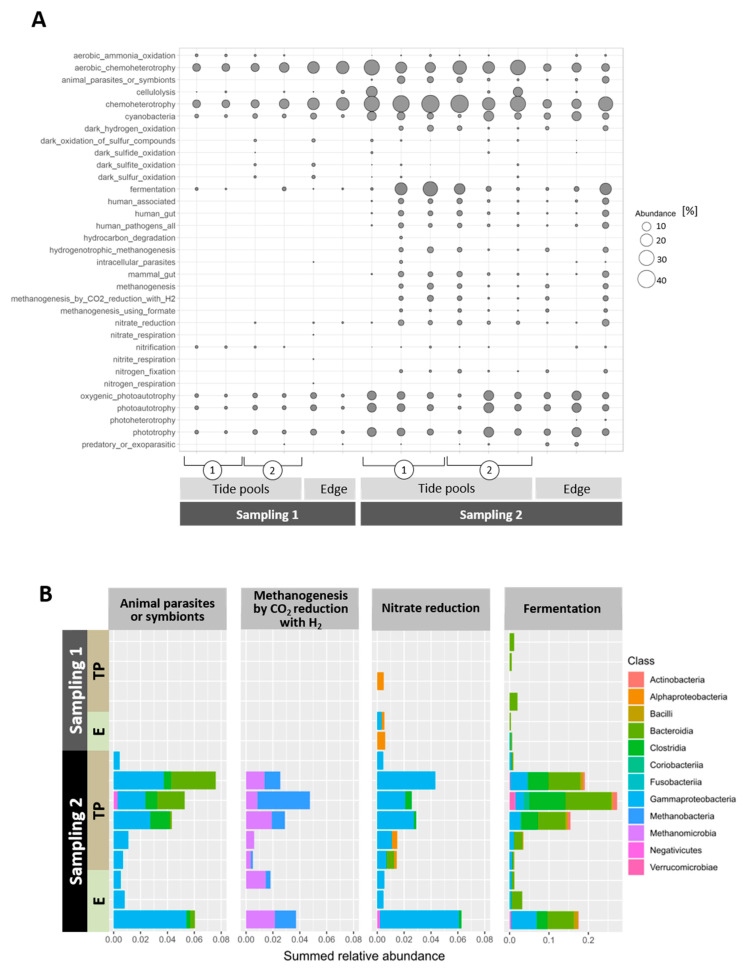
(**A**) Relative functional group abundance of ASVs associated with *Neogoniolithon* sp. surfaces from platform edge, tidal pool, and aquaria samples and their surrounding waters. ASVs were assigned to functional groups as described in Section 2. Abundance levels represent the sum of all ASVs assigned to a specific function. (**B**) Class level abundance of the main functional categories per study region and timing. TP = tidal pools; E = edge. Values represent the fraction from total ASVs identified per sample.

Taxonomic profiling of the microbiome provides information about “who is there” and reveals variations that may occur under various temporal and spatial scales. However, linking these variations to the microenvironment and the cellular functions is in most cases challenging. Based on an extensive literature survey and manual curation, Louca and colleagues combined experimental-based metabolic functions with relevant taxonomic groups [30], allowing them to obtain a presumptive functional annotation of ASV data (Figure 5A). Although this functional annotation does not provide a comprehensive picture, to our knowledge it is currently the best available for the Mediterranean marine system. Naturally, the most prominent general functions associated with the algal samples, throughout the study regions, are those related to basic metabolism, such as chemo-heterotrophy and fermentation. Where and when cyanobacteria were detected, their photosynthetic activity is noted as photo-autotrophy (Figure 5A).

To reveal the dynamic nature of spatial and temporal events underlying changes in functional groups, we identified the specific classes associated with some of the functional annotations for each study region and sampling time (Figure 5B). Noticeably, though fermentation capability is noted at all times, the nature of the classes performing it is sampling-time dependent (Figure 5B). Functional annotations reflecting the presence of anaerobic eubacteria and archaea in the tide pools, such as nitrate reduction, methanogenesis, and others corresponding to animal and human parasites and human guts (Figure 5), were recorded only in sampling 2.

## 4. Discussion

It is widely accepted that the microbiome composition and changes therein may serve as an indicator for the growth conditions of the relevant host [32,33,34,35]. We previously showed that despite its resilience and its wide distribution in the eastern Mediterranean rocky shore, *Neogoniolithon* sp. is quite sensitive to abiotic stress [1]. Previous studies on the roles played by CCA-associated microbiome (see Section 1), and the close proximity between bacterial cells and the algal thallus (Figure S1) prompted us to initiate studies on the nature of the bacteria associated with the alga. In future studies, this may serve to examine the bacterial impact on the algal fitness in the harsh conditions of the vermetid reefs. The first phase of the study, presented here, was to identify “who is there”. In this respect, it should be noted that relative abundance is not a measure of ecological or functional importance and that these should be assessed through studies of direct host–microbe interactions [36].

Although *Neogoniolithon* sp. is an important component of the eastern Mediterranean rocky shore environment, information on the microbial community associated with it in the Mediterranean is scarce [17]. We show that the *Neogoniolithon* sp. samples examined here host a distinct core community that is far more diverse than previously observed [17]. Further, this core community differs substantially from that observed in the water surrounding the algal samples in this study (Figure 2 and Figure 4) and in the aquaria cultures (Figure S1B). The four main phyla found to be associated with *Neogoniolithon* sp. were also identified in several other CCA microbiome surveys, despite being generated from a wide range of geographical locations, including the Caribbean Sea, the Great Barrier Reef, the South Atlantic Ocean, and the North Sea [14,16,37,38]. The relative abundance of Proteobacteria (49%), one of the “core members” in our samples (Figure 3 and Table S2), is lower than previously reported (97%) for Alphaproteobacteria associated with *N. brassica-florida* populations from the western Mediterranean basin [17]. Further, the second most abundant phyla observed here, Bacteroidota (25.2%), was not identified by Quéré et al. [17]. The reason(s) for the discrepancies between the two studies is unknown; it may reflect biotic or abiotic factors associated with the temporal and spatial characteristics of each study (e.g., western [17] and eastern (this study) parts of the Mediterranean Sea).

The abrasion platforms where the samples were collected from are a popular recreation site during spring and summer, hosting fishing, maritime sports and sailing activities from a small marina. In addition, the vermetid reefs of the eastern Mediterranean shore are often exposed to prolonged hot and dry eastern wind events during the spring and autumn seasons [39], leading to reduced circulation and water replacement in the pools. This may explain the effect of sampling timing on the microbiome associated with the *Neogoniolithon* sp. surface. The higher diversity observed in sampling 2 versus 1 edge samples (Figure 2) may also be attributed to the reduced circulation during springtime (where sampling 2 took place) and the development of a diversity of local conditions at the platform edge (which is constantly washed by waves during winter). Presence of sequences affiliated with anaerobic bacteria such as Clostridia (mainly Lachnospiraceae and Ruminococcaceae) and archaea involved in methanogenesis (Figure 4) in the tidal pools during sampling 2 but not in the surrounding water are just a few examples likely reflecting diverse conditions. It may be related to recreation activities during this time of the year; however, further sampling is required to support such conclusions. Though seasonality is likely to be an important driver of these distinct patterns, additional sampling during the year is required to establish more robust correlations, to be balanced with the need to minimize the damage to the protected abrasion platforms.

Several observations lend support to the possibility that the bacteria composition is affected by the local conditions in the reef, but detailed studies are needed to establish this notion. Examples include the presence of *Deinococci* bacteria (Figure 4). One of the members of this group, *Deinococcus radiodurans*, is considered the world’s toughest bacterium [40].

The high relative abundance of Bacteroidota (Figure 3 and Table S2) is more noticeable in association with the *Neogoniolithon* sp. surface than in the surrounding waters (Figure S6). Because they are abundant in various environments from animal guts to soil and marine habitats [41], they are often referred to as “environmental bacteria”, but the specific role of the various bacteria belonging to this group, if any, is yet to be established.

Taken together, though the interactions with prokaryotes likely play an important part in the algal life cycle and the ability of *Neogoniolithon* sp. to withstand the harsh conditions in the reef, it is yet to be experimentally revealed. This would require construction of a library of *Neogoniolithon* sp.-associated bacteria to test for specific interactions with the alga and their functional role. However, the observation that the bacterial community composition in laboratory (aquaria)-grown *Neogoniolithon* sp. differs from that observed in the field (Figure 1B) raises additional challenges. Given the limited access to field *Neogoniolithon* sp. Populations, interaction-directed studies may not be feasible in the field. A sophisticated aquarium is required to accurately simulate the harsh conditions of the vermetid reefs similarly to the environmental chamber constructed to study organisms inhabiting biological soil crust [42]. 

Finally, in an era where temperate coastal habitats are increasingly threatened by anthropogenic activities, a better understanding of the succession of the microbial community associated with *Neogoniolithon* sp. surfaces is essential to establish a deeper knowledgebase for this important reef builder. This may support the development and implementation of future conservation and monitoring strategies [43].

## Data Availability

Sequencing data (raw data) is publicly available via the European Nucleotide Archive-ENA with accession number PRJEB38881 under https://www.ebi.ac.uk/ena/browser/view/PRJEB38881 (Last access on 21 June 2021). Processed data and code are publicly available at GitHub: https://github.com/AlexanderBartholomaeus/ReefBuilderMicrobiome accessed on 21 June 2021.

## Supplementary Materials

The following are available online at https://www.mdpi.com/article/10.3390/microorganisms9071374/s1.
