# Supplementary material for: The Microbiome Associated with the Reef Builder Neogoniolithon sp. in the Eastern Mediterranean
Source: Microorganisms. 2021 Jun 24;9(7):1374. doi: 10.3390/microorganisms9071374 (PMC8306765; doi:10.3390/microorganisms9071374)
Supplement: Supplementary file 1 [file microorganisms-09-01374-s001.zip › SI PDFs/Figure S1.pdf]

Figure S1

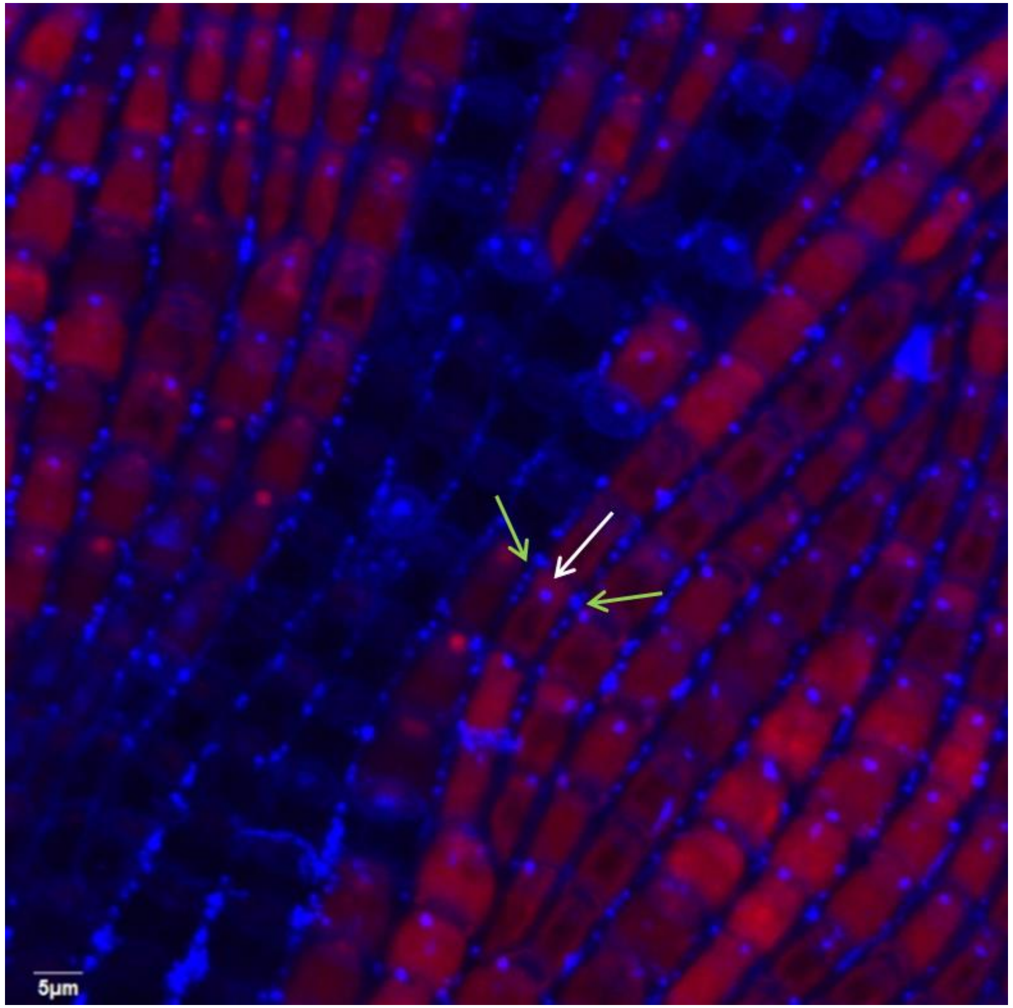

Figure S1: Confocal surface view image of *Neogoniolithon* sp. covered glass slide that shows DAPI-staining (blue) of bacterial cells (green arrows) and alga nucleus (white arrow). Algal covered glass slides were stained with DAPI (0.5 µg/mL) for 20 min, washed with DDW, and immediately mounted on a confocal microscope (FV-1200 microscope, Olympus, Japan) and analyzed with Olympus Fluoview software (version 4.0b). Excitation and emission for DAPI stained cells were 405 nm and 370 to 430 nm, and for chlorophyll fluorescence were 634 nm and 680 nm, respectively.
