# Supplementary material for: The Microbiome Associated with the Reef Builder Neogoniolithon sp. in the Eastern Mediterranean
Source: Microorganisms. 2021 Jun 24;9(7):1374. doi: 10.3390/microorganisms9071374 (PMC8306765; doi:10.3390/microorganisms9071374)
Supplement: Supplementary file 1 [file microorganisms-09-01374-s001.zip › SI PDFs/Figure S2.pdf]

Figure S2

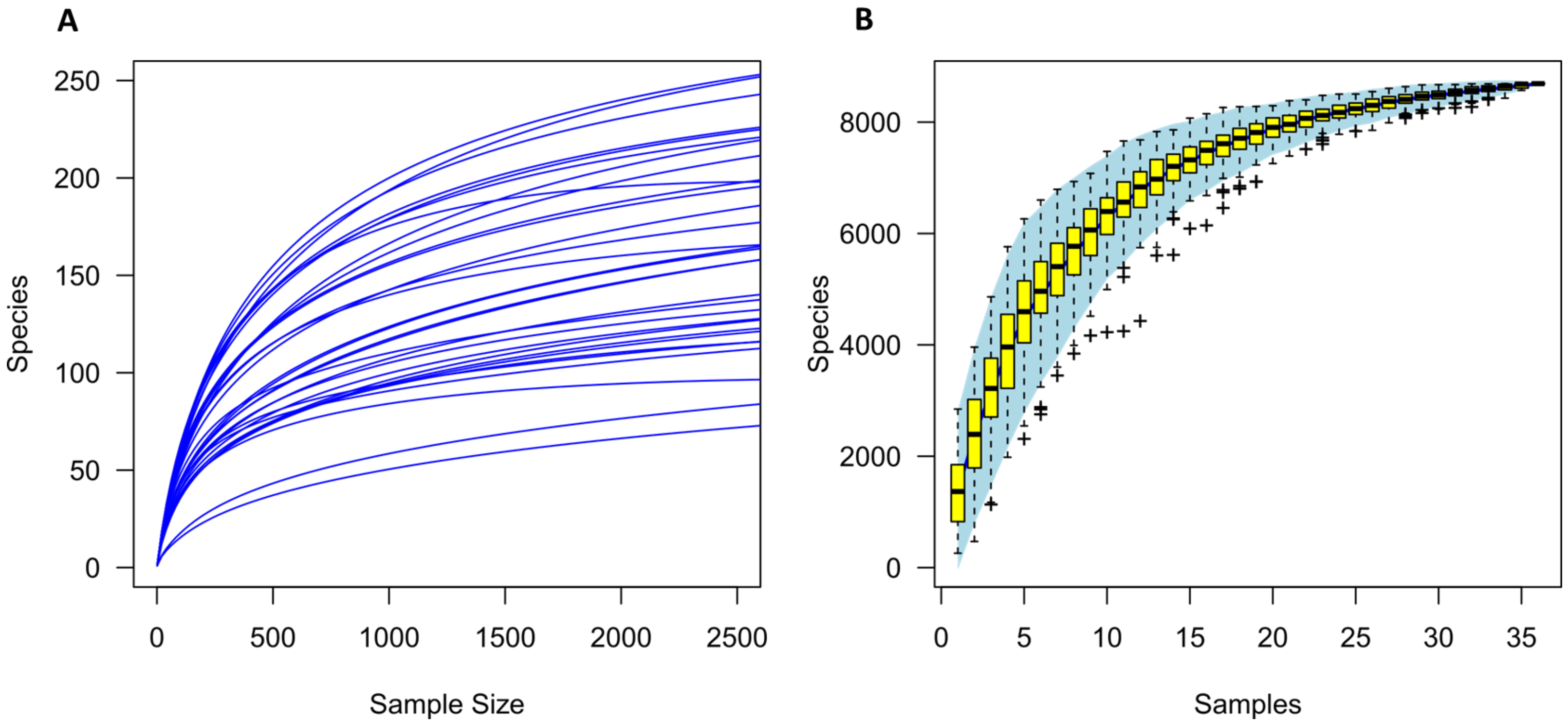

Figure S2: (A) Rarefaction curves. Number of species in relation to the sample size (sequencing depth; <2500 reads) reach a plateau for all samples, indicating a good representation of the most abundant taxa in the microbial community. (B) Species accumulation curve. The species accumulation in relation to the number of sequencing samples show a plateau starting from 20 samples indicating that sample replicates are enough to represent major species of the community.
