# Supplementary material for: The Microbiome Associated with the Reef Builder Neogoniolithon sp. in the Eastern Mediterranean
Source: Microorganisms. 2021 Jun 24;9(7):1374. doi: 10.3390/microorganisms9071374 (PMC8306765; doi:10.3390/microorganisms9071374)
Supplement: Supplementary file 1 [file microorganisms-09-01374-s001.zip › SI PDFs/Figure S3.pdf]

Figure S3

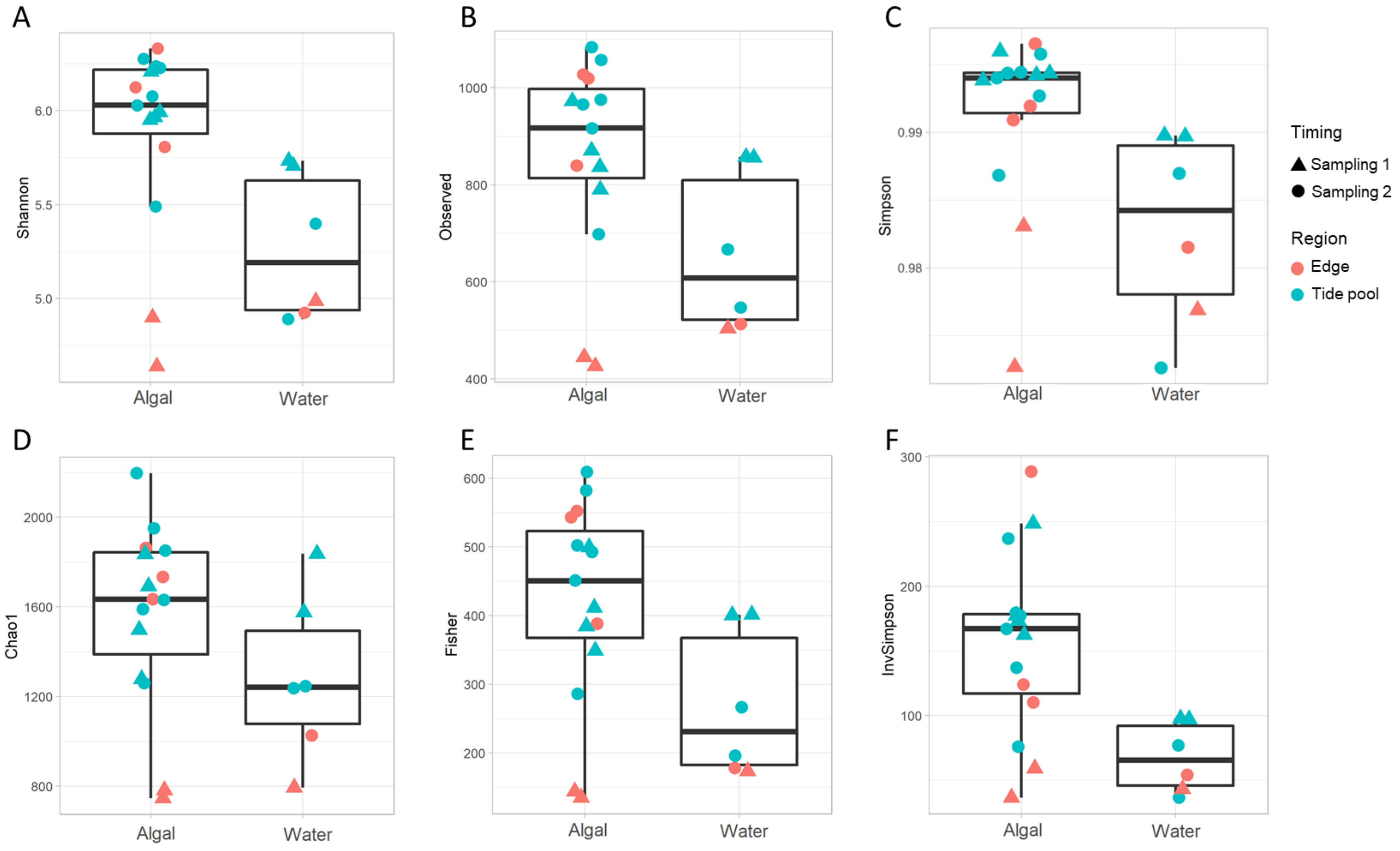

Figure S3: Alpha diversity analyses calculated using Shannon (A, as in Fig. 2 for comparison), Observed (B), Simpson (C), Chao1 (D), Fisher (E) and InvSimpson (F) indices for *Neogoniolithon* sp. surfaces and their surrounding water during samplings 1 (triangles) and 2 (circles), in different studying regions (Tidal pools, turquoise; platform edge, red).
