# Supplementary material for: The Microbiome Associated with the Reef Builder Neogoniolithon sp. in the Eastern Mediterranean
Source: Microorganisms. 2021 Jun 24;9(7):1374. doi: 10.3390/microorganisms9071374 (PMC8306765; doi:10.3390/microorganisms9071374)
Supplement: Supplementary file 1 [file microorganisms-09-01374-s001.zip › SI PDFs/Figure S4.pdf]

Figure S4

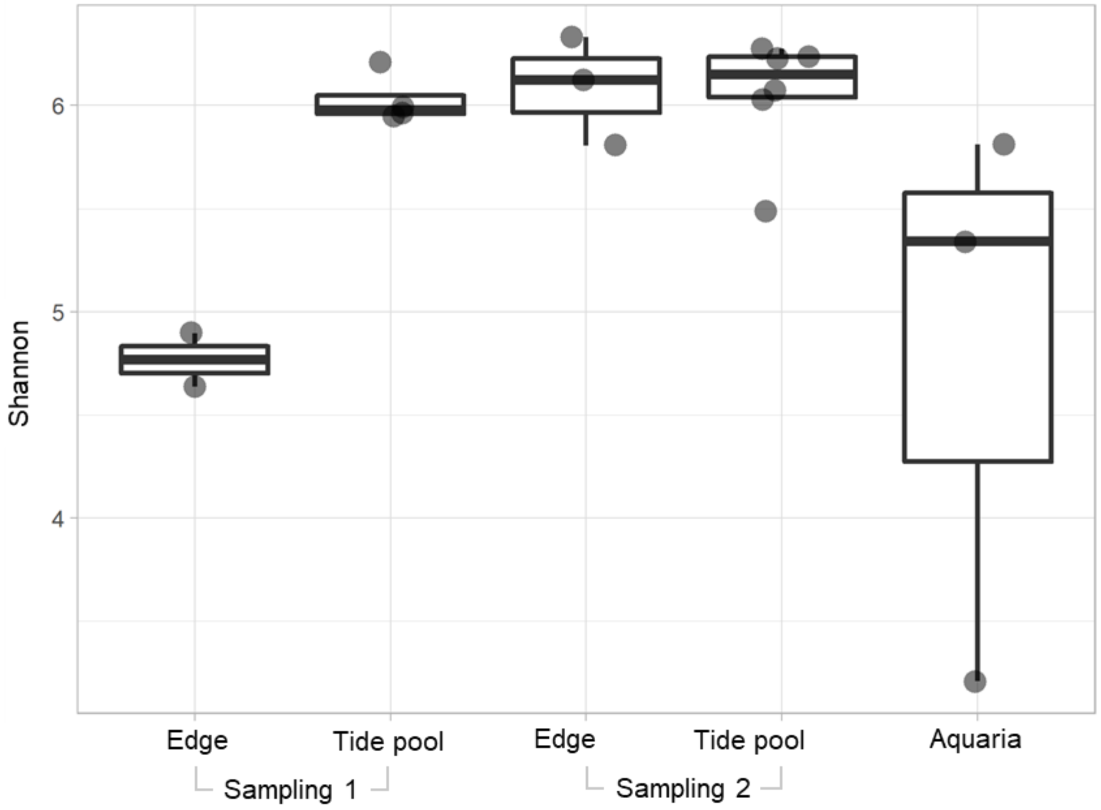

Figure S4: Alpha diversity analysis (calculated using Shannon index) of algal samples in tidal pools, platform edge (see Fig. 2B) and aquaria. In addition to crossings reported in Fig. 2B, significant difference was observed between aquaria and all other algal samples (P value = 0.0214).
