# Supplementary material for: The Microbiome Associated with the Reef Builder Neogoniolithon sp. in the Eastern Mediterranean
Source: Microorganisms. 2021 Jun 24;9(7):1374. doi: 10.3390/microorganisms9071374 (PMC8306765; doi:10.3390/microorganisms9071374)
Supplement: Supplementary file 1 [file microorganisms-09-01374-s001.zip › SI PDFs/Figure S5.pdf]

# Figure S5

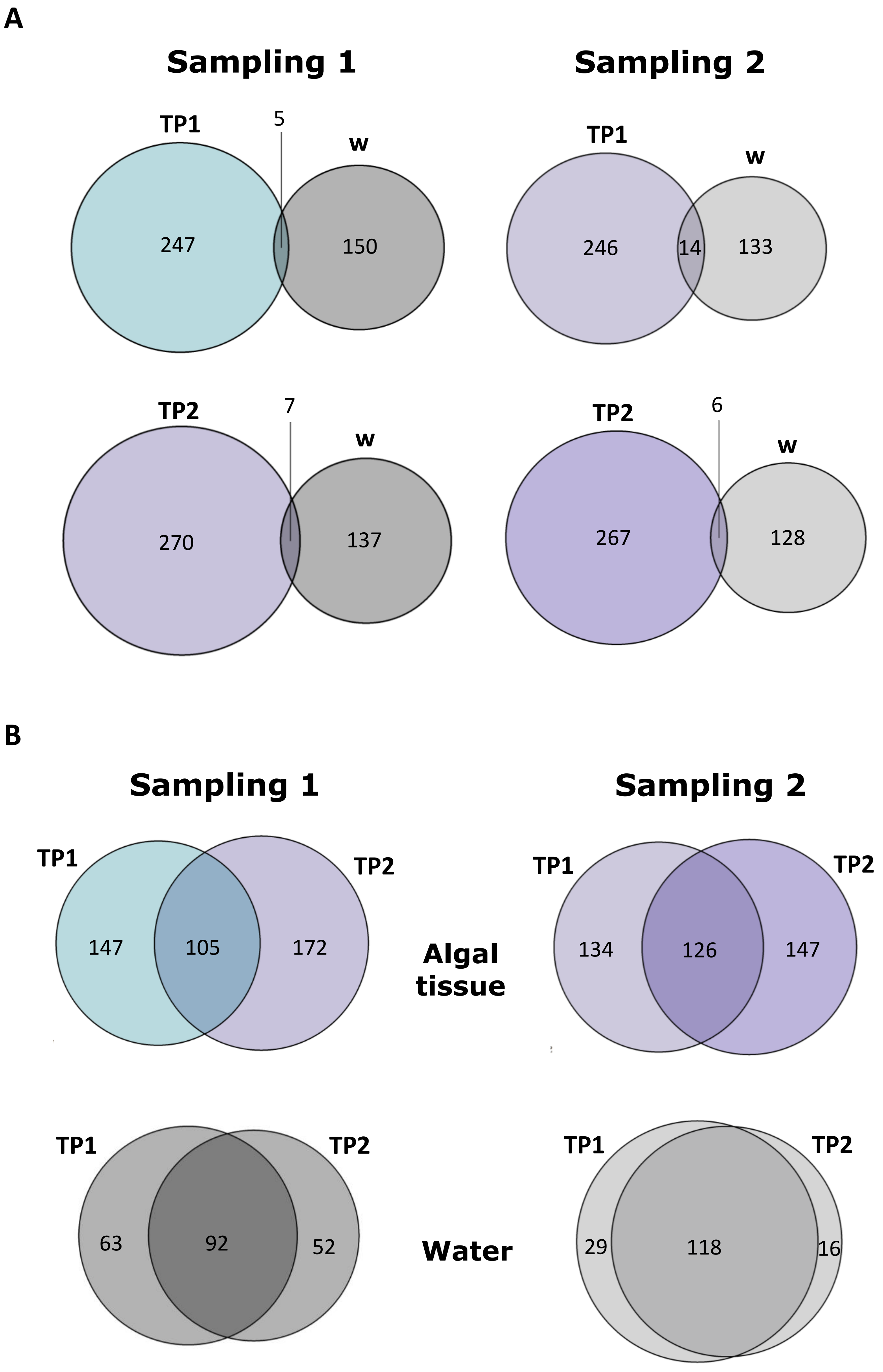


**Figure S5:** Venn diagram of shared ASVs between (A) *Neogoniolithon* sp. surfaces samples from (A) tidal pools (TP1, upper panel; TP2, lower panel) and their surrounding water, (B) algal tissue (upper panel) and their surrounding water samples (lower panel) in different tidal pools, in samplings 1 (left) and 2 (right).
