# Supplementary material for: The Microbiome Associated with the Reef Builder Neogoniolithon sp. in the Eastern Mediterranean
Source: Microorganisms. 2021 Jun 24;9(7):1374. doi: 10.3390/microorganisms9071374 (PMC8306765; doi:10.3390/microorganisms9071374)
Supplement: Supplementary file 1 [file microorganisms-09-01374-s001.zip › SI PDFs/Figure S6.pdf]

Figure S6

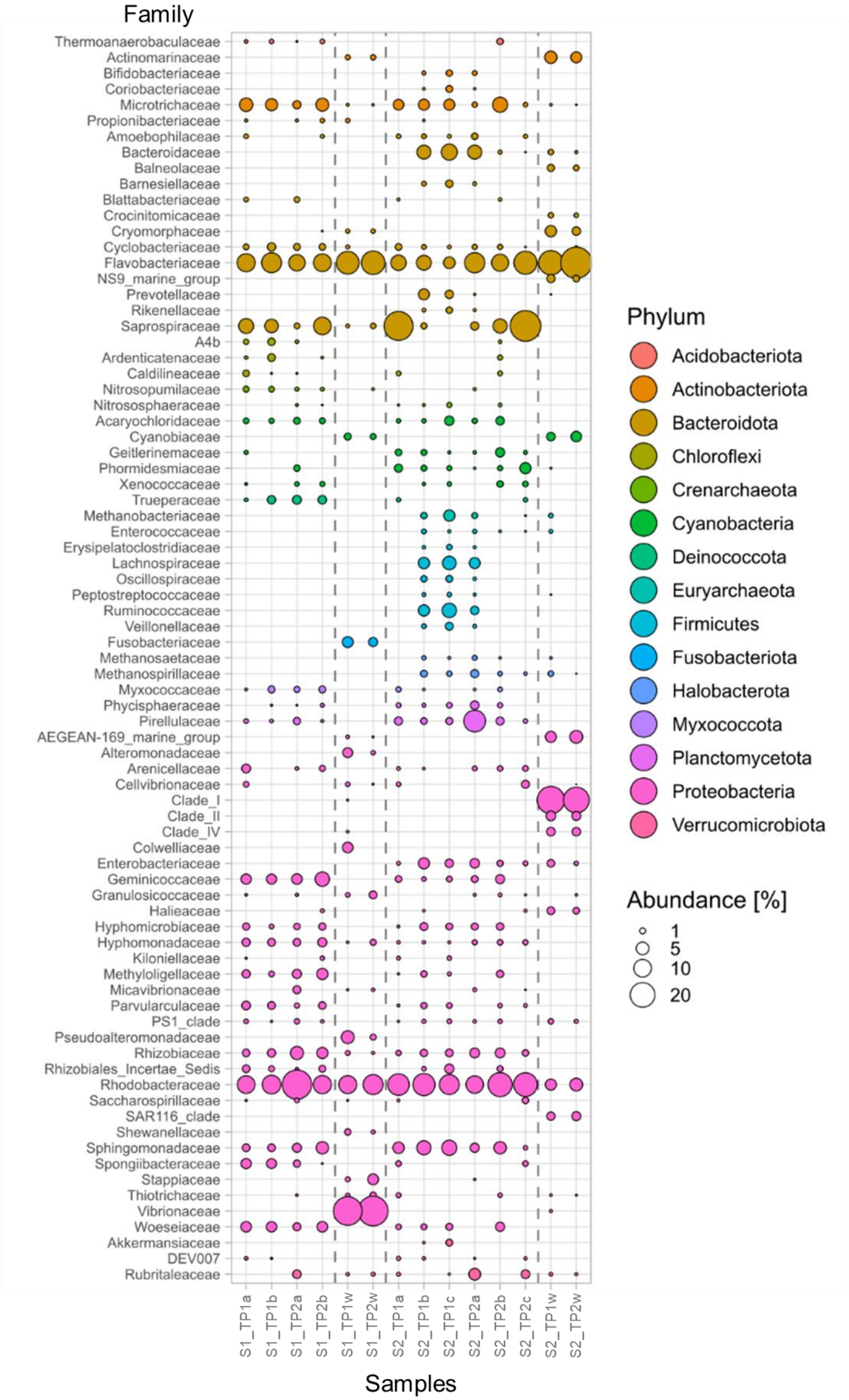

Figure S6: Relative abundance of ASVs associated with *Neogoniolithon* sp. surfaces in family taxonomic level from tidal pool and water samples in samplings 1 (n=2) and 2 (n=3). Colors indicate the different phyla. Bubble size represents the percentage from each family. See Figure 1 for the identity of the samples .S1 = sampling 1, S2 = sampling 2, TP (TP1 and TP2) = tidal pools, E = platform edge, w = water sample, a,b,c = biological replicates.
