# Supplementary material for: The Microbiome Associated with the Reef Builder Neogoniolithon sp. in the Eastern Mediterranean
Source: Microorganisms. 2021 Jun 24;9(7):1374. doi: 10.3390/microorganisms9071374 (PMC8306765; doi:10.3390/microorganisms9071374)
Supplement: Supplementary file 1 [file microorganisms-09-01374-s001.zip › SI PDFs/Legend to Supplementary Figures and Tables.pdf]

Legend to Supplementary Figures and Tables

Figure S1: Confocal surface view image of *Neogoniolithon* sp. covered glass slide that shows DAPI-staining (blue) of bacterial cells (green arrows) and alga nucleus (white arrow). Algal covered glass slides were stained with DAPI (0.5 μg/mL) for 20 min, washed with DDW, and immediately mounted on a confocal microscope (FV-1200 microscope, Olympus, Japan) and analyzed with Olympus Fluoview software (version 4.0b). Excitation and emission for DAPI stained cells were 405 nm and 370 to 430 nm, and for chlorophyll fluorescence were 634 nm and 680 nm, respectively.

Figure S2: (A) Rarefaction curves. Number of species in relation to the sample size (sequencing depth; <2500 reads) reach a plateau for all samples, indicating a good representation of the most abundant taxa in the microbial community. (B) Species accumulation curve. The species accumulation in relation to the number of sequencing samples show a plateau starting from 20 samples indicating that sample replicates are enough to represent major species of the community.

Figure S3: Alpha diversity analyses calculated using Shannon (A, as in Fig. 2 for comparison), Observed (B), Simpson (C), Chao1 (D), Fisher (E) and InvSimpson (F) indices for *Neogoniolithon* sp. surfaces and their surrounding water during samplings 1 (triangles) and 2 (circles), in different studying regions (Tidal pools, turquoise; platform edge, red).

Figure S4: Alpha diversity analysis (calculated using Shannon index) of algal samples in tidal pools, platform edge (see Fig. 2B) and aquaria. In addition to crossings reported in Fig. 2B, significant difference was observed between aquaria and all other algal samples (P value = 0.0214).

Figure S5: Venn diagram of shared ASVs between (A) *Neogoniolithon* sp. surfaces samples from (A) tidal pools (TP1, upper panel; TP2, lower panel) and their surrounding water, (B) algal tissue (upper panel) and their surrounding water samples (lower panel) in different tidal pools, in samplings 1 (left) and 2 (right).

Figure S6: Relative abundance of ASVs associated with *Neogoniolithon* sp. surfaces in family taxonomic level from tidal pool and water samples in samplings 1 (n=2) and 2 (n=3). Colors indicate the different phyla. Bubble size represents the percentage from each family. See Figure 1 for the identity of the samples .S1 = sampling 1, S2 =

sampling 2, TP (TP1 and TP2) = tidal pools, E = platform edge, w = water sample, a,b,c = biological replicates.

Table S1: DADA2 read count statistics during different levels of processing including sample classification.

Table S2: Abundance of various bacterial phyla associated with Neogoniolithon sp. surfaces. S1 = sampling 1, S2 = sampling 2, TP (1 and 2) = tidal pools, E = platform edge, w = water sample, a, b and c = biological replicates.

Table S3: Abundance of various bacterial classes belonging to the four main phyla shown in Table S2. S1 = sampling 1, S2 = sampling 2, TP (1 and 2) = tidal pools, E = platform edge, w = water sample, a, b and c = biological replicates.
